# Body Mass Index Perception, Body Image Dissatisfaction and Their Relations with Weight-Related Behaviors among University Students

**DOI:** 10.3390/ijerph16091541

**Published:** 2019-05-01

**Authors:** Hadia Radwan, Hayder A. Hasan, Haneen Ismat, Hala Hakim, Hiba Khalid, Leen Al-Fityani, Rawand Mohammed, Alzahraa Ayman

**Affiliations:** Department of Clinical Nutrition and Dietetics, College of Health Sciences, Sharjah Institute for Medical Research (SIMR), University of Sharjah, Sharjah 27272, United Arab Emirates; haidarah@sharjah.ac.ae (H.A.H.); nutri.haneen@gmail.com (H.I.); hala.hakim9@hotmail.com (H.H.); hiiba.khaliid@gmail.com (H.K.); leenrf@gmail.com (L.A.-F.); Rawand.alsaif17@gmail.com (R.M.); alzahraa_ayman@hotmail.com (A.A.)

**Keywords:** body image, body mass index, physical activity, perceived BMI, weight control, university students

## Abstract

The prevalence of obesity is increasing globally and is linked with poor body image perception. The aim of the present study was to identify the relationships of body image (BI) and body mass index (BMI) with weight control practices among university students. A cross-sectional study on a sample of 308 university students (150 males and 158 females) aged between 18 and 25 years was carried out. Face-to-face interviews were conducted based on a questionnaire that included socio-demographic, physical activity, the Figure Rating Scale (FRS) and body image dissatisfaction (BID) questions. The majority of the participants **(**81%: 58.2% females and 41.8% males) were dissatisfied with their BI. Females desired to lose weight and preferred diet to exercise, while males desired to gain weight and preferred exercise to diet (*p* < 0.001). About 56%, 39.5%, and 4.5% of the participants were normal, overweight/obese, and underweight, respectively. There was a significant, strong correlation (*R*^2^ = 0.84, *p* < 0.001) between perceived BMI and actual BMI. Actual BMI showed a significant strong correlation with the BID (*r* = 0.57, *p* < 0.001). The results highlighted the need to increase awareness of the importance of healthy eating behaviors and regular physical activities to improve body size, shape perception, and satisfaction among college males and females.

## 1. Introduction

It has been noted that there has been a drastic increase in the attention given to the study of the relationship between body image (BI) and obesity [1,2]. Meanwhile, the concepts of overweight and obesity, as defined by body mass index (BMI), are not well understood by many people. Several researchers have reported misperceptions of weight status by adults [3,4]. Exploring an individual’s perception of his/her weight status and relating this perception to his/her real weight can help in determining the unrealistic views of BI [5], which is defined as the individual’s perceptions, thoughts, and feelings about his or her body [6]. Research studies have reported that awareness of being overweight or obese is an essential factor to start following weight-related behaviors [7]. Consequently, a key to health and weight control is proper self-weight perception and body satisfaction. Body size is usually over- or underestimated [8]; that could be due to various factors that play a role in making up the concept of BI, such as psychological elements, socio-cultural influences, friends, family members, age, and gender [9]. The scanty of existing evidence indicated that imprecise weight perceptions among overweight and obese persons are associated with weight-related attitudes (like eating and weight concern) and weight-related behaviors (like fewer weight loss attempts, unhealthful dietary intake, and lower physical activity levels), which are the key components that inhibit weight loss and maintenance [10,11]. Lynch et al. stated that obese women who perceived themselves as obese attempted to lose weight, while obese women who perceived themselves as being normal or overweight gained weight [12]. This indicates that more realistic body size perceptions are associated with less weight gain.

In theory, individuals who feel dissatisfied with their BI are more likely to engage in behaviors to fight the discomfort [13], but the literature has proven that a negative BI might also lower the incentive to practice physical activity [14]. Studies have investigated perceptions of the terms ‘overweight’ and ‘obese’ through a variety of methods [15,16]. Previous research reported that women are more likely than men to misperceive their weight. It was found that 29% of normal weight women reported that they perceived themselves as overweight compared to only 8% of men who wrongly perceived their weight [17].

There is a growing body of evidence that obesity is connected to poor BI, particularly among women with overweight and obese, who report greater body image dissatisfaction (BID) than normal weight women [1]. Attitudes against fat and obesity inclinations were reported among university students [18]. The social environment of the students in university settings heightens the attention toward social norms associated with appearance and allure, which may increase the risk of students utilizing unhealthy body-change modalities [19].

Kuan et al. utilized the Stunkard Figure Rating Scale (FRS) and stated that the greater part of the females in a Malaysian university favored an underweight figure as their optimal model, whereas about 30% of the males picked an overweight figure as their optimal model [20]. In general, females were progressively more worried about body weight, body shape, and dietary patterns than males. BID may avert people from taking part in healthy lifestyle activities such as exercise [21]. Heinberg et al. suggested that those who are moderately dissatisfied with their body images might be spurred to reduce weight or improve wellbeing status by changing their eating and physical activity regimens [22]. Significant improvements were observed in the BI of obese women who achieved an average weight loss of 22 kg [23]. Therefore, it is essential to identify the person’s perceived BID, which could be a predictive factor of subsequent overweight or obesity and eating disorders [24]. The key question is how to identify those in need of BI intervention so that programs can be integrated for weight loss treatment. Early diagnosis of one’s awareness of being overweight or obese was shown to be an essential factor for successfully losing weight in both men and women [7]. Few studies have discussed BID and related factors in the United Arab Emirates (UAE). It was reported earlier that 74.8% of female university participants were dissatisfied with their body image [25]. Another study concluded that 73% (78% females and 58% males) were dissatisfied with their body image [26]. Identifying body image dissatisfaction among university students, who are a high-risk group, as the college environment can cause high stress and concerns related to body image, might be helpful for early intervention programs in terms of helping students to improve their perceptions about their body weight and image and thereby prevent unhealthy eating behaviors.

Therefore, this study was designed to identify body image dissatisfaction and its association with real and perceived body mass index among university students as well as its relation to weight-related behaviors (diet and physical activity).

## 2. Methods

### 2.1. Study Design

A cross-sectional study was carried out at the University of Sharjah in the UAE.

### 2.2. Study Population and Sample Size

Participants were recruited using a convenience sampling method. Convenience sampling was used in order to meet the sample size requirement within the stipulated data collection period. Thus, a convenient sample of 308 university students aged between 18 and 25 years and of Arabic origin was approached and recruited from the three campuses at the university. After being consented, the students were interviewed face to face, and then their anthropometric measurements were taken (weight and height) according to usual standards. Any participant who was above the age of 25 years, non-Arab, and did not fill the questionnaire or did not give full anthropometric measures was excluded from the study.

### 2.3. Tools

#### 2.3.1. Questionnaires

The questionnaire included socio-demographic information (e.g., age, gender, marital status), a physical activity questionnaire (the International Physical Activity Questionnaire (IPAQ)), and the Figure Rating Scale (FRS) adapted from Stunkard et al. [16].

#### 2.3.2. The Figure Rating Scale (FRS)

Self and body sizes were assessed by the use of the Stunkard FRS. It consists of nine silhouettes ranging from very thin (a value of 1) to very obese (a value of 9) [16]. Participants were asked to rate how they perceived their current body shape—or “how they look”—by choosing a score that corresponded to their figure on a scale ranging from 1 to 9. The participants were also required to state the ‘ideal’ figure they desired—or “how they would like to look”. The discrepancy between the two figures is seen as an indication of dissatisfaction.

#### 2.3.3. Body Image Dissatisfaction (BID)

A body image dissatisfaction (BID) variable was created by subtracting the participant’s current body size FRS score from the ideal body size FRS score. A BID score ≥ 1 was considered to indicate that a participant “desired to be thinner”; a BID score < 1 was considered to indicate that a participant “desired to be heavier”; a BID score of zero was considered to indicate that a participant was satisfied with his or her body.

#### 2.3.4. Diet-Related Practices

In the questionnaire, participants were asked if they followed a diet to either lose or gain weight and about the type of diet they followed.

#### 2.3.5. Physical Activity Questionnaire 

A reliable and valid short form (seven questions) of the International Physical Activity Questionnaire (IPAQ) [27] was used to assess each participant’s physical activity pattern. Each participant was requested to indicate the type, frequency (days per week), and duration (hours or minutes per day) of each physical activity he/she performed amid the last seven days. The assessment is based on the intensity of physical activities categorized as vigorous (e.g., aerobic walking, jogging, and running), moderate (e.g., brisk walking, general home exercises, recreational swimming), and just normal walking. The level of physical activities is classified as low, moderate, and high based on metabolic energy (MET)-minute per week. MET for walking is 3.3, for moderate activity is 4.0, and for vigorous activity is 8.0.

#### 2.3.6. Anthropometric Measurements

After the participants completed the self-administered questionnaire, their body weight (kg) and height (cm) were measured by using Seca 220 Telescopic Measuring Rod for Column Scales for height/ weight measurements (Seca, Hamburg, Germany). Participants were measured with their clothing was on, shoes removed, and pockets emptied.

Actual BMI (kg/m^2^) was calculated by dividing weight (kg) by the height squared (m). BMI was classified based on World Health Organization (WHO) classifications [28]: underweight (BMI < 18.5), normal weight (BMI 18.5–24.9), overweight (BMI 25–29.9), and obese (BMI > 30). Moreover, participants were asked to report their weight and height then their perceived BMI was calculated accordingly.

### 2.4. Data Analysis

Data were analyzed using Statistical Package for the Social Sciences (SPSS) version 22.0 (SPSS, Chicago, IL, USA). Data are presented as frequency and percentages. Chi-square test was used to study the relationships between the categorical variables. Differences were considered significant at *p* < 0.05.

### 2.5. Ethical Considerations

The study protocol was approved by the Research Ethics Committee (REC) at the University of Sharjah (REC-17-04-06-01-S). An informed consent form was signed by the participants in which they were assured of confidentiality and anonymity and that their participation was voluntary.

## 3. Results

A total of 308 students from the University of Sharjah aged 18 to 25 years (<20 years; 49% and ≥20 years; 51%) participated in this study: One hundred and fifty-eight (51.3%) were females and one hundred and fifty (48.7%) were males. The majority were single (97.7%), from majors not related to health (54.6%). The actual BMI of more than half of the participants was normal (56%); similarly, the majority of the participants (57.3%) perceived their BMI as normal. Most of the participants were physically active (73.1%) and reported that they were not following any diet (54.6%). About 80.9% of the total sample were dissatisfied with their body image, with more than half of them wanting to be thinner (Table 1). 

**Figure 1:**
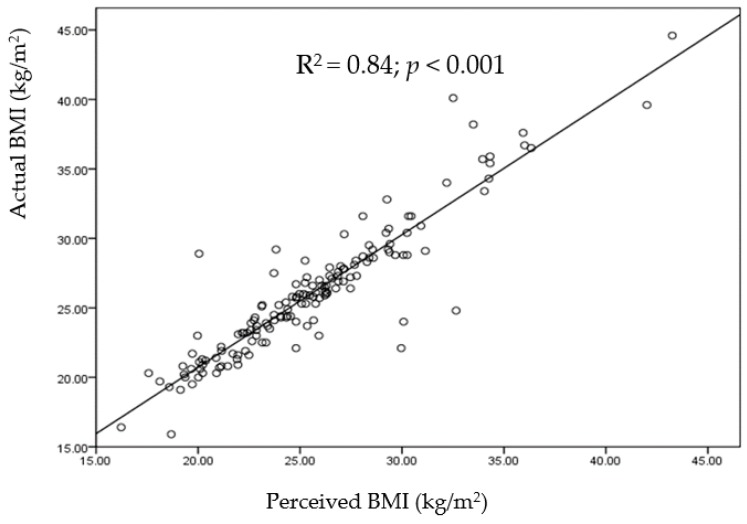
Relationship between perceived BMI and actual BMI.

There was a significant, positive, strong correlation (*R*^2^ = 0.84, *p* < 0.001) between perceived BMI and actual BMI, as shown in Figure 1. Moreover, the actual BMI showed a highly significant strong correlation with the BID (*r* = 0.57, *p* < 0.001). In general, the majority of the participants in all BMI categories perceived their BMI correctly. Table 2 shows that perceived BMI is significantly dependent on the actual BMI(*X^2^ =*546,P< 0.001). Most of the participants who perceived their BMI as normal actually had normal BMI (91.3%; 158/173). However, only 3.5%, 2.9%, and 2.3% of those who perceived their BMI as underweight, overweight, and obese respectively were actually of normal BMI. While 75.6% (65/86) of overweight participants correctly identified themselves as overweight, 19.8% of them reported being of normal BMI. The majority of obese students perceived themselves as obese (80%), whereas 17.1% and 2.9% of them perceived their BMI as overweight and normal, respectively, as shown in Table 2. 

Table 3 demonstrates that the majority of the participants were dissatisfied with their body shape (249/308; 80.9%), with almost similar distribution among males (120/150; 80%) and females (129/158; 81.6%). However, the direction of BID was significantly different between male and female participants. More than half of the females (59.3%) wanted to be thinner, whereas the majority of males (66.7%) desired to be heavier (*p* < 0.001). Moreover, more than half of the female participants reported that they followed a diet (61.4%) and 75% of them did not do any physical activity, while the majority of the male students (56.2%) were physically active, and only 38.6% of them followed a diet. 

Table 4 shows that more than half of the female participants who had a BID score ≥ 1 (desire to be thinner) reported that they followed a diet (54.3%); however, 62.9% did not do any physical activity. On the other hand, the majority of the male students (88.9%) who desired to be thinner exercised without following any diet (55.6%). With regard to participants who desired to be heavier, the majority of them stated that they did not follow a diet (79.2%, 85.4% females and males, respectively) and did not do any physical activity (62.5%, 83.3% females and males, respectively).

## 4. Discussion

The aim of this study was to examine the relationships between perceived and actual BMI, body image dissatisfaction, and weight-related behaviors among university students. The results of this study reflected the university students’ thoughts about their body shape and weight. This could help in avoiding future health problems arising from current and ideal body shape misconceptions and could contribute to raising awareness on healthy weight status in order to achieve body shape satisfaction.

Generally, in this study, most of the participants perceived their BMI accurately, as reported by Som and Mukhopadhyay, who found a moderate agreement between the actual and perceived weight of Indian urban adolescent girls [29]. However, another study among undergraduate university students in Pakistan showed a high prevalence of weight misperception (42.2%) [30]. Also, about one-third of female university students in Karachi misperceived their weight [31].

An interesting finding in this study is that although the prevalence of overweight and obesity among the study participants was low, a high rate of BID was observed. This might be explained by the idea that BID reflects the subjective component of one’s body image and the degree of satisfaction with one’s own body size or specific body parts [32], while BMI reports the physical body measurement of a subject’s weight and height. This is of particular concern, since BID has been found to be closely related to eating disorders [33,34]. Thus, investigating one′s realistic perception and self-awareness of one’s own body and not only relying on BMI figures can provide valuable information and an additional tool for clinical settings dealing with weight management and eating disorders. However, this study revealed that there were discrepancies between actual and ideal body images among the participants, as the majority of both males and females were dissatisfied with their body images. The prevalence of this dissatisfaction was higher than what was reported by Ferrari et al., who found about 70% of the university students in Brazil were dissatisfied with their body shape [35]. In the current study, however, the pattern of dissatisfaction among our participants significantly varied between the two genders. Among those who desired to be heavier, the majority were males, while those who were aspiring to be thinner were mostly females. This finding is in line with previous research documenting a great dissatisfaction among females, where males desired to be larger and females desired to be smaller [36]. This is probably because females were more likely to choose a thinner body shape than males to represent males’ perception of female attractiveness. Moreover, it was assumed that males desired to gain weight in order to have a muscular appearance [37]. 

In the current study, there was a significant gender difference in BID as well as participants’ choice for weight control practices. Female students who wanted to be thinner preferred following a diet for weight loss, whereas for males who desired to be heavier, the majority were physically active and less likely to follow a diet. Hence, male students were exercising more frequently than females. Females in the UAE are known to have low physical activity levels due to social norms and lack of facilities [38,39]. Adding to this, a possible reason for low physical activity among females in this study may be related to their low body satisfaction, which discouraged their participation in physical activity. Neumark-Sztainer et al. showed that the lower the body satisfaction, especially among females, the lower the physical activity levels [40]. Moreover, it has been reported that body image concerns can serve as obstacles to participating in physical activity among adolescents girls [41]. Another study from the United State revealed that those who were not happy with their weight demonstrated lower levels of physical movement in comparison with satisfied individuals [42]. El Ansari et al. reported that dissatisfied females were more likely to follow a diet to lose weight, and they were at a higher risk of developing eating disorders [43]. Wang et al. revealed that adolescent females were more likely to express weight dissatisfaction than young males, and body weight perception and dissatisfaction were related to their weight control practices [44].

Notwithstanding the health advantages associated with an active lifestyle, most of the dissatisfied students at the university were not involved in inadequate levels of physical activity. This is in concurrence with previous research hypothesizing that BID is associated with smaller likelihood of participating in physical activity in both women and men [14]. Previous research reported that overweight perception is a cognitive barrier to physical activity among women and men with excess weight [45]. In contrast to our findings, a study among university students in Saudi Arabia reported that no significant difference was found between body shape satisfaction and physical activity status among the students [46]. Hence, higher education institutes provide a good opportunity to reach a large number of students and to encourage healthy eating behaviors and proper weight perceptions. Previous reports have reported that university students may be considered a high-risk group for eating disorders because a higher proportion of them tend to have body image concerns and follow unhealthy eating behaviors and attitudes [19,47].

## 5. Conclusions

The study highlights the importance of focusing on body image dissatisfaction among both females and males, with body image dissatisfaction associated with fewer attempts to either diet or exercise. Despite the low prevalence of overweight and obesity among university students in the present study, a high rate of BID was noted. It is essential to further comprehend the components that are related to the BID among the female and male students and to confirm whether they are promoters or inhibitors of dieting and/or exercising. Intervention programs should simultaneously attempt to increase physical activity and improve body satisfaction, especially among females, and this should be investigated further to delineate the reasons for the females not engaging in physical activity to lose weight. 

Universities should screen and identify students who are at risk by measuring not only their BMI but also their body image perception. Awareness and education programs should be disseminated in academic settings to promote healthy weight concept and improve recognition of proper body image perception among all students. Health professionals should address the unrealistic body image concerns and the important stressors of the high-risk students by focusing on their healthy eating behaviors, healthy food choices, portion control, and regular physical activities.

### Limitations

Several limitations of this study were recognized, such as the small sample size, which may not be representative of the whole population and limits generalizability. Additionally, self-reported data can contain several potential sources of recall bias, which could affect the accuracy of our results. A limitation for the short form of the IPAQ questionnaire is that it has a tendency to overestimate physical activity levels when compared to some other questionnaires and to the long version of the IPAC. As a cross-sectional study, the findings are correlations, not causations, with the inability to decide the direction of the effects.

## Figures and Tables

**Table 1:** General characteristics of the participants (*N* = 308). BMI: body mass index; BID: body image dissatisfaction.

Variables		*N*	%
Gender	Males	150	48.7
	Females	158	51.3
Age	<20 years	152	49%
	≥20 years	156	51%
Marital Status	Single	301	97.7
	Married	7	2.3
Actual BMI	Underweight	14	4.5
	Normal weight	173	56.0
	Overweight	86	28.0
	Obese	35	11.5
Perceived BMI	Underweight	19	6.1
	Normal weight	177	57.3
	Overweight	76	24.6
	Obese	36	11.7
BID	Satisfied	59	19.1
	Desire to be heavier	72	23.4
	Desire to be thinner	177	57.5
Major	Health	129	41.9
	Non- health	179	58.1
Follow diet	Yes	109	35.4
	No	199	54.6
Physical Activity	Inactive	83	26.9
	Active	225	73.1

**Table 2:** Self-perceived BMI and actual BMI among university students (*N* = 308) using chi-square test.

Actual BMI	Perceived BMI				*p* Value
	Underweight % (*N*)	Normal % (*N*)	Overweight % (*N*)	Obese % (*N*)	
Underweight	92.9 (13)	7.1 (1)	0.0 (0)	0.0 (0)	< 0.001
Normal	3.5 (6)	91.3 (158)	2.9 (5)	2.3 (4)	
Overweight	0.0 (0)	19.8 (17)	75.6 (65)	4.7 (4)	
Obese	0.0 (0)	2.9 (1)	17.1 (6)	80.0 (28)	

**Table 3:** Body image dissatisfaction (BID) and weight-related behaviors by gender among dissatisfied participants (*N* = 249) using chi-square test.

Variables		*N*	Gender		*p*	Odds Ratio	95%Confidence Interval	
			Male (*N* = 120) % (*N*)	Female (*N* = 129) % (*N*)			Low	High
BID	Desire to be thinner (BID ≥ 1)	177	40.7 (72)	59.3 (105)	<0.001	0.34		
	Desire to be heavier (BID ≤ 1)	72	66.7 (48)	33.3 (24)			0.19	0.61
Follow Diet	Yes	101	38.6 (39)	61.4 (62)	0.012	1.92	1.14	3.20
	No	148	54.7 (81)	45.3 (67)				
Physical Activity	Active	185	56.2 (104)	43.8 (81)	<0.001	3.85	2.03	7.27
	Inactive	64	25(16)	75 (48)				

**Table 4:** Distribution of body image dissatisfaction (BID) and weight-related behaviors by genders.

BID Category	Female (*N* = 158)				Male (*N* = 150)			
	Diet		Physical Activity		Diet		Physical Activity	
	Yes (%)	No (%)	Yes (%)	No (%)	Yes (%)	No (%)	Yes (%)	No (%)
Desire to be thinner (BID ≥ 1)	54.3 (57)	45.7 (48)	37.1 (39)	62.9 (66)	44.4 (32)	55.6 (40)	88.9 (64)	11.1 (8)
Satisfied (BID = 0)	10.3 (3)	89.7 (26)	48.3 (14)	51.7 (15)	16.7 (5)	83.3 (25)	83.3 (25)	16.7 (5)
Desire to be heavier (BID ≤ 1)	20.8 (5)	79.2 (19)	37.5 (9)	62.5 (15)	14.6 (7)	85.4 (41)	83.3 (40)	16.7 (8)
